# The control of branching morphogenesis

**DOI:** 10.1098/rsob.130088

**Published:** 2013-09

**Authors:** Dagmar Iber, Denis Menshykau

**Affiliations:** 1 Department of Biosystems Science and Engineering (D-BSSE), ETH Zürich, Basel, Switzerland; 2 Swiss Institute of Bioinformatics (SIB), Basel, Switzerland

**Keywords:** branching, computational modelling, *in silico* organogenesis

## Abstract

Many organs of higher organisms are heavily branched structures and arise by an apparently similar process of branching morphogenesis. Yet the regulatory components and local interactions that have been identified differ greatly in these organs. It is an open question whether the regulatory processes work according to a common principle and how far physical and geometrical constraints determine the branching process. Here, we review the known regulatory factors and physical constraints in lung, kidney, pancreas, prostate, mammary gland and salivary gland branching morphogenesis, and describe the models that have been formulated to analyse their impacts.

## 2. Introduction

**Figure 1.**
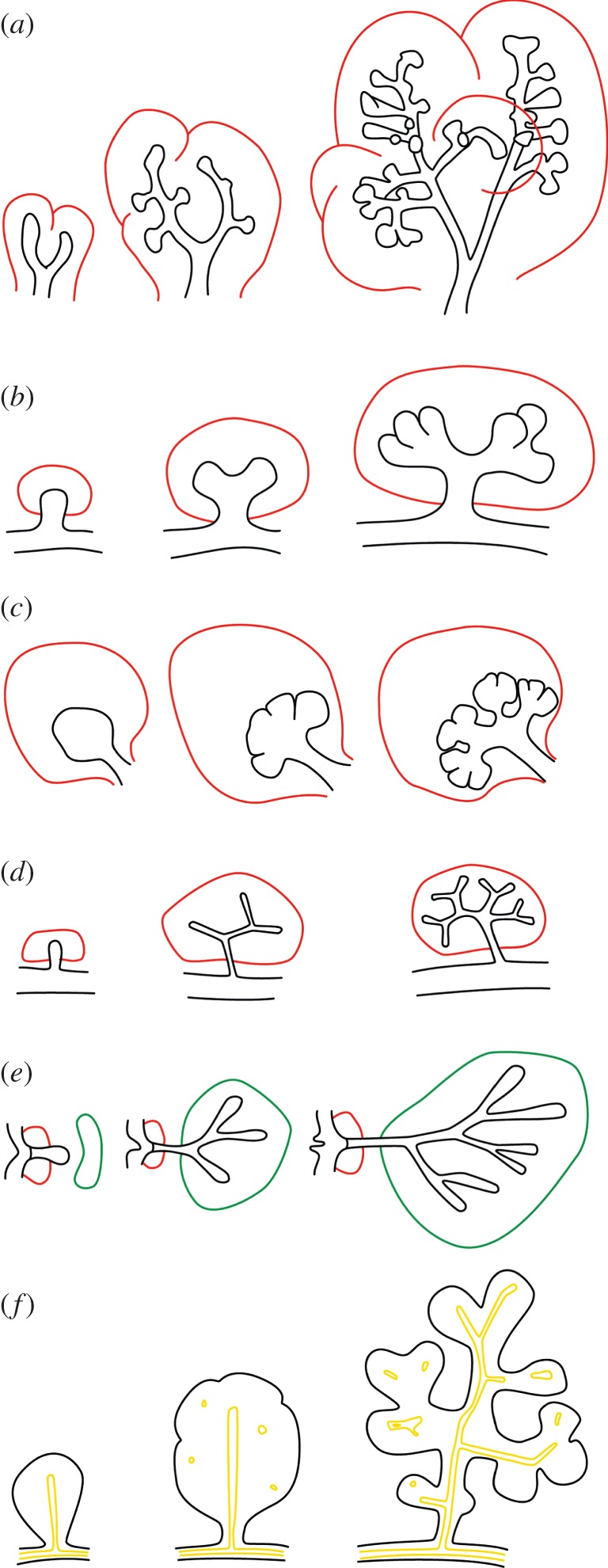
Branching morphogenesis. Typical branching pattern over developmental time in the (*a*) lung, (*b*) ureteric bud, (*c*) salivary gland, (*d*) prostate, (*e*) mammary gland and (*f*) pancreas. The epithelium is shown in black, the mesenchyme in red, the fat pad in the mammary gland in green and the lumen in the pancreas in yellow.

**Figure 2.**
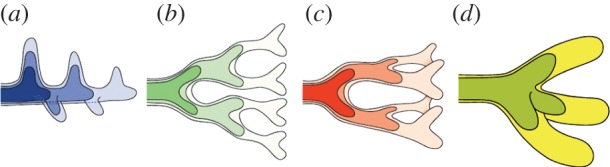
Modes of branching: (*a*) lateral branching, (*b*) planar bifurcation, (*c*) orthogonal bifurcation and (*d*) trifurcation. (*a*–*c*) Reproduced from [1].

Branching morphogenesis is observed in many organ systems (figure 1) and in many different species. The branching process in the mammalian lung (figure 1*a*) and in its analogue in flies, the trachea, has been studied in particularly great detail. The bronchial tree arises from the sequential use of three geometrically simple modes of branching: domain branching (figure 2*a*), planar bifurcation (figure 2*b*) and orthogonal bifurcation (figure 2*c*) [2]. Trifurcations (figure 2*d*) have also been documented in the lung [3], but these are much more prevalent in the ureteric bud of the kidney (figure 1*b*); in the kidney bifurcations and trifurcations dominate, at the expense of lateral branching [4–6]. Similar modes of branching are observed also in glands (figure 1*c–f*). In this review, we will focus on branching during mammalian organogenesis, but ignore neuronal and vasculature branching as well as branching observed in plants. For reviews of these branching systems, we refer to [1,7–10].

**Figure 3.**
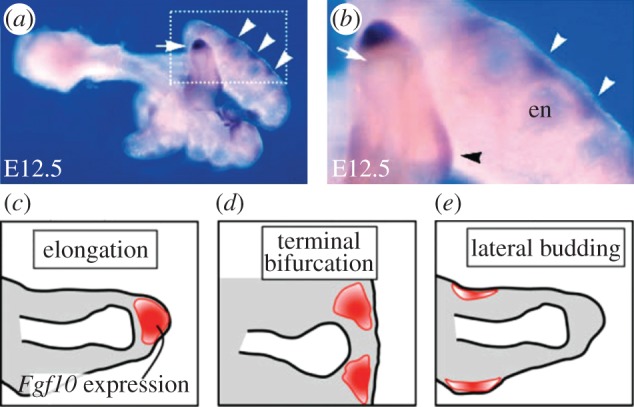
FGF10 in the developing lung. (*a,b*) *Fgf10* expression at embryonic day (E) 12.5. High expression levels of *Fgf10* are observed in the distal mesenchyme of the tip (white arrow), as well as on the sides of the tips of a bud (white arrowheads). (*b*) High magnification of the white dotted box in (*a*). Note that *Fgf10* expression is absent in the mesenchyme adjacent to the endoderm (en) of the tip. Localized *Fgf10* expression is also observed in the mesenchyme around the stalk (black arrowhead). (*c–e*) Schematic of the spatial distributions of *Fgf10* expression in (*a*,*b*). *Fgf10* is expressed in the red region. The white area indicates the lumen, the grey area the mesenchyme, the black line in between the epithelium. The outer black lines mark the mesothelium. The three types of spatial distributions of *Fgf10* expression generate different branching modes: (*c*) elongation, (*d*) terminal bifurcation and (*e*) lateral budding. The entire figure is adapted from Hirashimi & Iwasa [11]; (*a,b*) adapted from the original publication of Bellusci *et al.* [12].

Most processes in higher organisms are intricately regulated by a network of signalling factors. To arrive at a branched structure, these signalling components would have to form a pattern in space that precedes bud outgrowth, as is indeed observed in the case of fibroblastic growth factor (FGF) 10 in the developing lung bud (figure 3*a,b*). The concentration of FGF10 at the distal tip of the lung bud would then direct elongation (figure 3*c*), whereas terminal branching would be the result of a split localization of FGF10 (figure 3*d*), and lateral branching would result from FGF10 signalling being restricted to spots on the side (figure 3*e*).

Such patterns can, in principle, emerge from a pre-pattern that goes back to earlier phases of embryonic development or that arises spontaneously from regulatory interactions. A number of theoretical models have been developed to explain how patterns can emerge and how these are read out in space. We will review these concepts in the context of their use in the models for branching morphogenesis.

**Figure 4.**
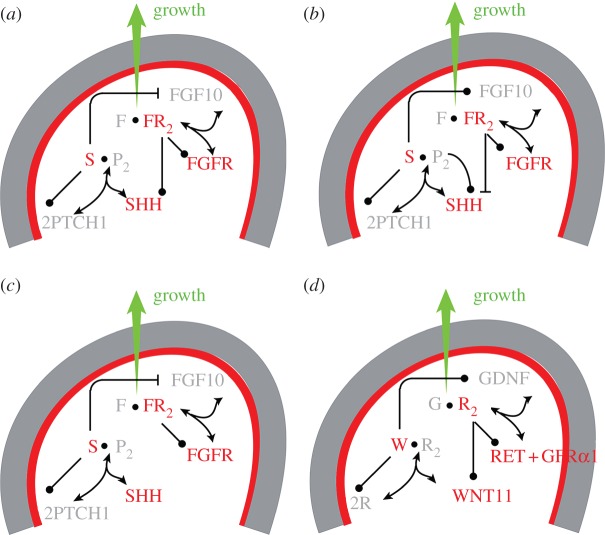
Signalling networks in branching morphogenesis. The core signalling networks that have been described to regulate branching morphogenesis in (*a*) lung and prostate, (*b*) salivary gland, (*c*) pancreas and (*d*) kidney are shown. In the lung, prostate, salivary gland and pancreas FGF10 (F) signalling directs outgrowth of the epithelium. *Fgf10* is expressed in the mesenchyme (grey) and binds to its receptor (R) in the epithelium (red). FGF10-bound receptor not only directs outgrowth, but also regulates expression of *Shh* (S) ((*a*) upregulation in the lung and prostate, (*b*) downregulation in the salivary gland, (*c*) no reported regulation in the pancreas). SHH binds its receptor PTCH1 (P) and the SHH-receptor complex, in turn, regulates *Fgf10* expression ((*a,c*) downregulation in the lung, prostate and pancreas, (*b*) upregulation in the salivary gland). All ligand–receptor signalling also upregulates the expression of the receptor. (*d*) In the case of the ureteric bud, GDNF (G) induces bud outgrowth and GDNF-receptor binding stimulates expression of the receptor *Ret* and of *Wnt11* (W) in the epithelium. WNT11, in turn, causes upregulation of *Gdnf* expression in the mesenchyme.

While the branching patterns are similar overall, there are important differences (figure 1), and the signalling proteins that control the branching programme in the different organ systems are not always the same (figure 4). Thus, FGF10 appears to drive outgrowth of lung buds [12], prostate [13] (both figure 4*a*), salivary glands [14,15] (figure 4*b*) and pancreatic buds [16,17] (figure 4*c*), whereas ureteric bud outgrowth is controlled by the transforming growth factor (TGF)-β family ligand glial-derived neurotrophic factor (GDNF; figure 4*d*) [18–21], and no single growth factor has yet been defined for the mammary gland [7], though FGF receptor 2 is known to be required for ductal elongation in mammary glands [22]. These signalling proteins are controlled by further proteins, and the regulatory networks differ between organs. Given these differences in the signalling networks, mechanisms based on the interplay of physical forces have also been explored.

**Figure 5.**
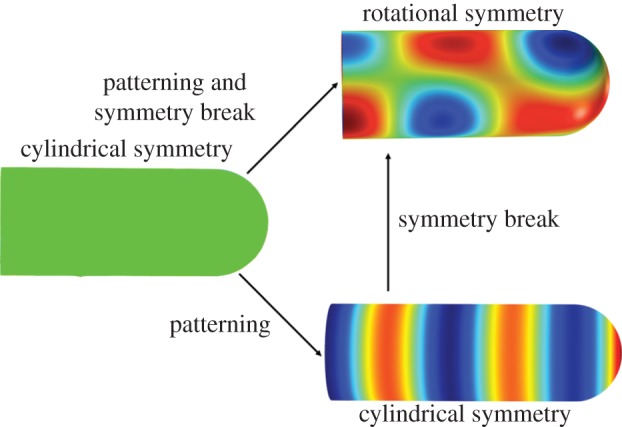
Patterning and symmetry break. A cylinder with a homogeneous morphogen concentration exhibits cylindrical symmetry. Patterning mechanisms may introduce stripes or spots. The spotty cylinder exhibits rotational symmetry, and such pattern would support the outgrowth of defined branches.

Key to all such mechanisms is an instability that results in a patterning and/or a symmetry-breaking event. The elongating tube can be regarded as a cylinder with a cap (figure 5). A cylinder with a homogeneous distribution of morphogens on its surface has a cylindrical symmetry, such that if the cylinder is rotated by any angle along the main axis, then the pattern will not change (figure 5). During branching morphogenesis, a symmetry-breaking event must occur, because the outgrowing bud has approximately cylindrical symmetry, whereas the branching events (i.e. bifurcations, trifurcation or lateral branching) change the cylindrical symmetry into a rotational symmetry. Symmetry breaking is a fundamental process, and occurs many times and at various scales during embryo development, as recently reviewed in the collection of *Cold Spring Harbor Perspectives in Biology* (for the editorial, see [23]).

Computational models can help us to explore the impact of the signalling interactions, physical forces and domain geometries, and thus discern a minimal set of rules and interactions from which the observed pattern can emerge. In the following, we will discuss the different models that have been applied to branching morphogenesis of various organs. We will be interested, in particular, in how far similar mechanisms can explain branching morphogenesis in different organ systems.

## 3. Lung branching morphogenesis

Early lung branching morphogenesis is stereotyped, and the lung tree arises from the sequential use of three geometrically simple modes of branching: domain branching, planar bifurcation and orthogonal bifurcation [2]. Transitions from one mode to another are restricted to four routines and lead to three defined sequences that are used to build the entire lung tree [1,2]. In the domain branching mode, the lung bud elongates, and new buds appear first on one side of the stalk in a direction perpendicular to the main axis of the cylinder and subsequently on the another side of the stalk. Planar and orthogonal bifurcations represent two consecutive rounds of bifurcations, and differ in the second round of branching, which occurs in the same plane in the case of planar bifurcations, and orthogonal to the first plane in the case of orthogonal bifurcations [2]. Trifurcations (figure 2*d*) have also been documented recently [3]. The domain branching mode is used to build the backbone of the respiratory tree, whereas planar bifurcations form the thin edges of the lobes, and orthogonal bifurcations create the lobe surfaces and fill the interior. Only few variations and errors are observed in wild-type littermates [2,3]. The regularity of the process implies that the branching process is not random, but tightly controlled by genetically encoded information. It is an open question how a stereotyped branching architecture with millions of branches can be encoded with only a few genes.

### 3.1. Fractals

**Figure 6.**
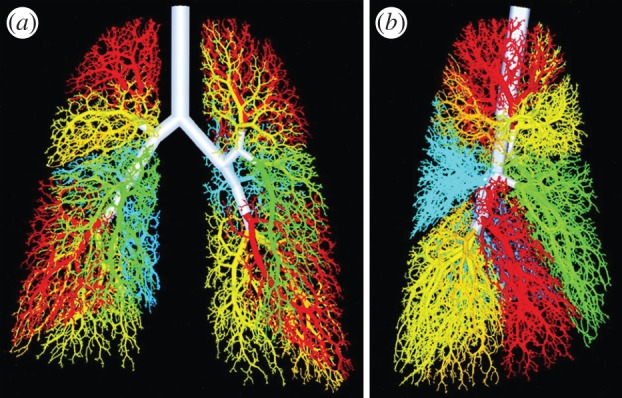
A three-dimensional fractal model of an airway tree with 54 611 branches; branches distal to different segmental bronchi are shown in same colour as segmental bronchus. (*a*) Anterior view and (*b*) right lateral view. The figure and legend were adapted from Kitaoka *et al.* [24].

Fractals are complex structures that can be formed by the repetitive application of a set of simple rules. If the lung was a fractal structure, then its complex architecture could result from a set of simple rules, as has been demonstrated in many fractal models of the lung. The most sophisticated and accurate of these models was created by Kitaoka *et al.* [24], who required nine basic and four complementary rules to fill the three-dimensional thoracic cavity with a branched tree that very much resembled that of the lung (figure 6). So is the lung a fractal-like structure?

In the lung, dichotomous branching gives rise to two daughter branches with smaller diameter than the mother branch. To obtain a fractal series, the shrinkage factor would need to be the same in all generations.

**Figure 7.**
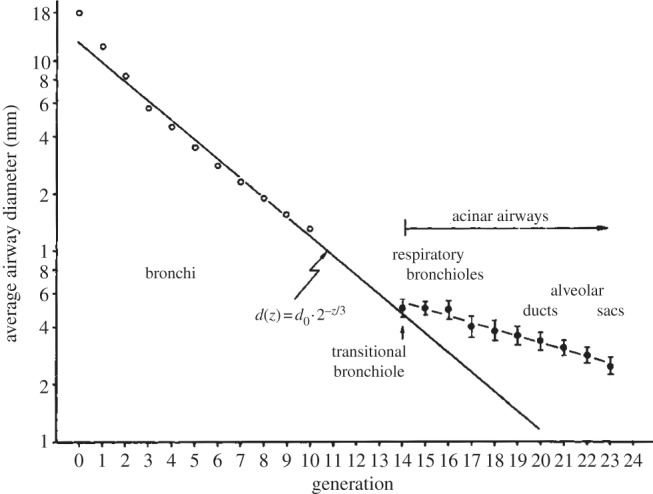
A fractal-like organization of the lung. A logarithmic plot of the airway branch diameter against the branching generation reveals two exponential laws with different scaling factor for conducting and respiratory airways. For the conducting part, the relation *d*(*z*) = *d*(0) × 2^–*z*/3^ was observed for the diameters *d* in generation *z*. For such shrinkage factor in the diameter, the total volume of all branches remains constant for dichotomous branching and the entropy generation during breathing is minimal [27]. The plot is adapted from Bleuer [28].

Both the length and the diameter of lung branches have been determined for the adult lungs of several mammals [25]. The ratio between diameter and length differs between species, but it is conserved in a given species (i.e. length/diameter ∼ 3 in humans) [26]. We can therefore focus on the diameter. Measurements show that the diameter *d_z_* of the lung branches in generation *z* decreases exponentially with the branching generations (figure 7). More formally we can write3.1
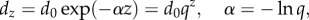
where *q* = exp(–*α*) < 1 is a constant scaling factor. We can rewrite this equation as
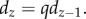

The diameter and length in each subsequent generation thus decreases by a constant factor *q* < 1. Such constant scaling law reflects the scale invariance of fractal patterns (i.e. the pattern looks the same no matter what scale of the structure we zoom in to). The geometrical figures thus repeat themselves at progressively smaller scales as characteristic for self-similar fractals.

Mathematically, fractals are defined as any series for which the Hausdorff dimension (a continuous function) exceeds the discrete topological dimension. Topologically, dimension corresponds to the number of independent coordinates that are required to describe the position of an object in that particular space. To illustrate the Hausdorff dimension consider a square. If we scale down each side by a scale factor *r* = 1/2, then we require 2^2^ squares to fill the original square. With *r* = 1/*n*, we require *n*^2^ squares. In three dimensions, we would require *n*^3^ cubes. More generally, if we scale down each side by scale factor *r* = 1/*n*, then we require 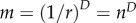 elements to fill the original space, where *D* is the dimension:
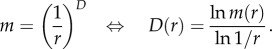


Branching in the lung is dichotomous, and from one generation to the next *m* = 2 elements thus emerge. The diameter is scaled by *q* = exp(–*α*) = 2^–1/3^ in the first 10–15 generations that generate the conducting airways (i.e. for the airways that do not participate in gas exchange; figure 7). Accordingly, the fractal dimension of the lung airways is
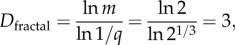
which is greater than the topological dimension of the branching sequence, and which implies that the airway tree fills the entire three-dimensional thoracic space in which it is embedded. Fractal properties in nature have remained controversial because, unlike in mathematical sequences, the geometrical figures are observed over a small, finite scale (number of generations), and fitted scaling factors are therefore not particularly reliable [29]. In fact, a different scaling factor is observed for the lower, respiratory branches (figure 7, acinar airways), and attempts to simultaneously fit both series have led to a different scaling factor [30], and to the suggestion of a power-law relation [25].

There is, however, independent strong support for the fractal-like properties of the branching structure of the conducting airways. Thus, the diameter relation *d_z_* = 2^–*z*/3^ *d*_0_ in the conducting part of the airways has been shown to result in an airway architecture that permits breathing with minimal energy wastage (entropy generation) by minimizing the combined effects of dead volume and resistance [27]. Thus, the upper part of the lung is dead volume because it must be ventilated without contributing to gas exchange with the blood. The larger the diameters in this part of the structure, the larger the dead volume, and thus the more energy is wasted in each breath. However, shrinking these diameters results in an increase in air flow resistance. The diameter relation *d_z_* = 2^–*z*/3^*d*_0_ is the one that minimizes the combined contributions of these two effects. The fractal-like organization of the conducting parts of the bronchial tree thus permits the generation of a space-filling, energy-efficient architecture for ventilation, where all the tips have similar distances from the origin of the airways in the trachea, based on a simple set of rules. While this may explain why such a bronchial tree evolved, it does not reveal the driving force during development, because the embryo does not breathe, and the developing lung is filled with fluid rather than air. Moreover, the diameters and lengths in early lung development do not yet exhibit a fractal-like pattern. So what are the mechanisms that regulate branching morphogenesis?

### 3.2. Mechanical models

**Figure 8.**
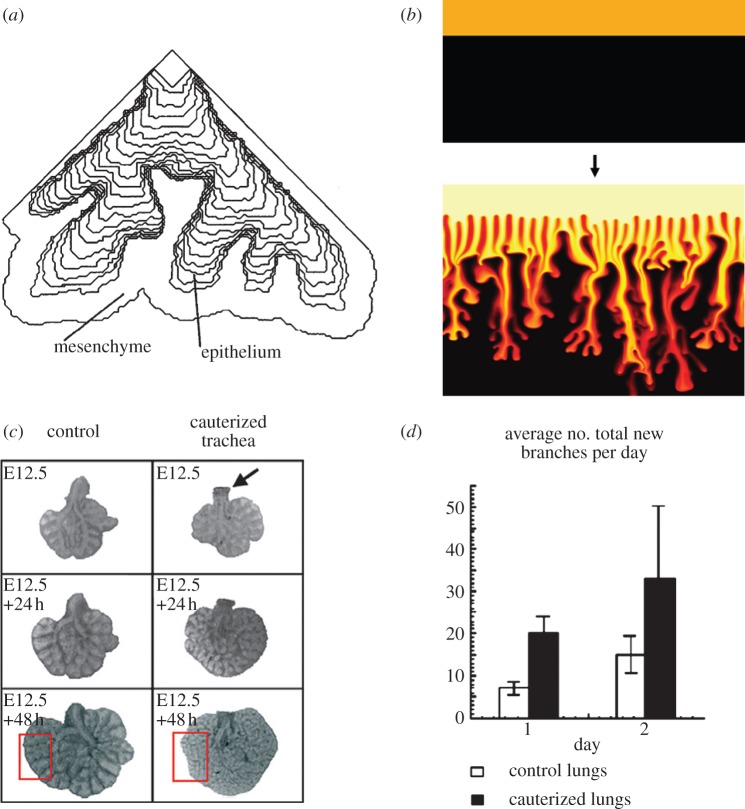
Branching as a result of mechanical differences. (*a*) Branching driven by a viscosity difference between the fluid in the lumen and the mesenchyme; adapted from Lubkin & Murray [33]. (*b*) Viscous fingering is observed in a system with two immiscible fluids of differing viscosities; adapted from Jha *et al.* [34]. (*c*,*d*) The impact of internal pressure on branching; adapted from Unbekandt *et al.* [35].

Culture experiments demonstrate that the mesenchymal tissue largely defines the branching pattern when mesenchymal tissue and epithelial tissue of different organs are combined. One striking example came from tissue recombination experiments in which lung mesenchyme induced branching of the ureteric bud with a pattern characteristic of lung epithelium (i.e. with increased lateral branching) [31]. Mesenchymal tissue that either induced or did not induce branching of an epithelial layer in culture was found to differ in its mechanical properties, though the exact differences have not been defined [32]. In line with these observations, lung branching was proposed to be driven by the difference in the viscosity of the luminal/amniotic fluid and the mesenchyme, separated by a ‘skin’ of surface tension, the epithelium [33]. Two fluids with different viscosities exhibit an instability if the more viscous liquid pushes the less viscous one in response to an external force (figure 8*a*); in physics, this effect is often referred to as viscous fingering (figure 8*b*) [34,36]. This effect, though physically plausible, has more recently been recognized as biologically incorrect by the authors [37,38], because (i) branching morphogenesis can proceed also without mesenchyme, (ii) branching morphogenesis can proceed without growth (hence without growth pressure), and (iii) the robustness of the branching process suggests that the branching process is highly controlled, which would not be the case for a near-equilibrium instability.

In subsequent studies, the authors studied a similar model, but interpreted the two (Stokes) fluids as epithelium and mesenchyme, with surface tension in between and focused on the effects of an external ‘clefting force’, which was assumed to mainly originate from traction forces in the mesenchyme (though epithelial forces were considered as well) [38,39]. The analysis showed that the higher the relative viscosity of epithelium and mesenchyme, the wider the resulting clefts and the faster the clefts emerge [38,39]. How the position of the clefting force would be controlled has not been explored, but this could, of course, be regulated by the signalling networks that control branching morphogenesis.

Signalling factors play a key role in branching morphogenesis, and branching of the epithelium in the absence of mesenchyme is observed only if the appropriate signalling factors (i.e. FGF in the case of the lung) are added to the Matrigel [40]. Similarly, while increased internal pressure is a mechanical effect that leads to an increase of lung branching in *in vitro* cultures (figure 8*c,d*), this effect also depends on the activity of FGF signalling, is not observed in *FgfR2b* null mice and is reduced in *Fgf10* hypomorphic lung explants [35]. Signalling networks thus appear to be at the core of the control of branching morphogenesis, and seem to both affect and to be affected by mechanical properties of the tissue. Thus, signalling networks regulate the hydraulic pressure during lung organogenesis [41] as well as the actomyosin-mediated contractility of cells, and local differences in the extracellular matrix (ECM). Inhibition of the actomyosin-mediated contractility in lung explants decreases branching [42], whereas activation of the contractility increases branching [43]. The impact of the ECM structure on branching morphogenesis has previously been reviewed [44].

### 3.3. Signalling models

The core signalling module that controls branching morphogenesis in the lung comprises the two diffusible proteins, FGF10 and SHH (figure 4*a*); mutations in both genes and their downstream effectors abrogate branching morphogenesis [45–47], whereas mutations in other genes at most modulate the branching process. FGF10 has been shown to induce the outgrowth of lung buds [12]. FGF10 and SHH engage in a negative feedback loop, in that FGF10 signalling induces *Shh* expression in the epithelium, whereas SHH signalling represses *Fgf10* expression in the mesenchyme.

#### 3.3.1. Diffusion-limited growth

**Figure 9.**
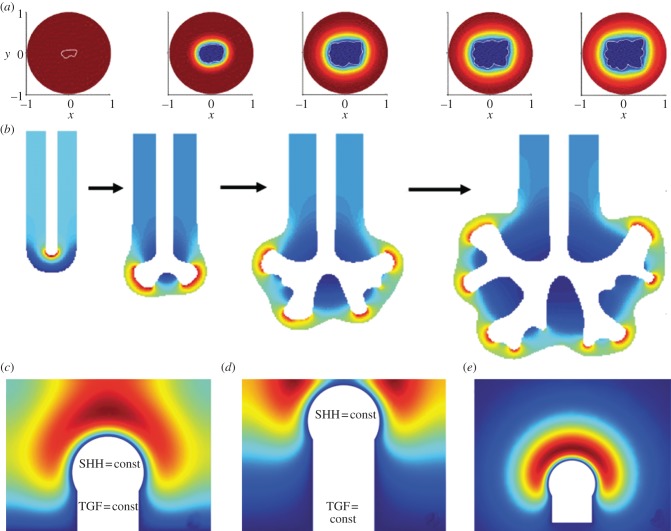
Patterning models based on the local variations in the distance of the epithelium from the source of FGF10. (*a*) Branching as a result of diffusion-limited growth. Tissue (delimited by white line) that is placed into a solution with a low concentration of an outgrowth-inducing signalling factor (such as FGF10 in the lung) will degrade the signalling factor and thus induce a concentration gradient (red, high; blue, low). As a result of small irregularities in the tissue shape (white line), some part of the tissue will be closer to the higher concentration and accordingly start to grow out faster, thus experiencing even higher (relative) concentrations; adapted from Hartmann & Miura [48]. (*b*) With FGF10 produced mainly in the submesothelial mesenchyme and its receptor produced only in the epithelium, an FGF10 gradient can be expected to emerge. If the FGF10 concentration is homogeneous close to the mesothelium and on the epithelium, then the gradient would be steeper, the shorter the distance between epithelium and mesothelium. If cells read out gradients rather than concentration, then small differences in the distance could trigger self-avoiding outgrowth of branches; adapted from Clément *et al.* [49]. (*c–e*) Distance-based mechanism based on SHH and FGF10. SHH is produced only by the epithelium, and represses *Fgf10* expression at high concentrations. Accordingly, *Fgf10* expression can be expected to be lower, the closer are epithelium and mesothelium [12]. (*c*) Assuming that SHH induces *Fgf10* expression at low concentrations, the FGF10 concentration is high as long as the bud is sufficiently far away from the boundary, thus supporting bud elongation. (*d*) As the bud approaches the impermeable boundary, the FGF10 profile splits, thus supporting bifurcating outgrowth. (*e*) The bifurcation in (*d*) requires an impermeable boundary and is not observed on an open domain. The FGF10 distribution in (*c*–*e*) was calculated according to the model presented by Hirashima & Iwasa [11]. (*c*–*e*) Adapted from Menshykau *et al.* [50].

The emergence of branches in cultures of mesenchyme-free lung epithelium has been proposed to be the result of diffusion-limited growth (figure 9*a*) [48,51,52]. The diffusion-limited regime of growth is observed when the concentration of the growth factor inducing tissue growth is low, and degradation of the growth factor can therefore result in sharp concentration gradients. Small protrusions (which are closer to the source) will then experience higher morphogen concentrations. As a consequence, these protrusions will grow faster and thus get even closer to the source. In the experiment, mesenchyme-free lung explants were placed into a Matrigel-containing FGF. The lung tissue degrades FGF and thus acts as a sink. At sufficiently low FGF concentrations, a gradient of FGF emerges with a minimum concentration of FGF in the proximity of the epithelium and maximum concentration away from the lung explant. For such low concentrations, irregular growth of the explant is indeed observed (figure 9*a*). In the case of high concentrations of FGF in the Matrigel, no such gradient can emerge, and a uniform expansion of the lung explant was observed [48]. When the conditions of diffusion-limited growth were met, both the model and the experiments show that the mechanical strength of the cytoskeleton can suppress only branching. In the chick lung, a branched structure is formed only dorsally, whereas a cyst structure (air sac) is formed ventrally during development. Miura *et al.* [53] suggest that this difference can be accounted for by differences in the FGF10 diffusivity that would be sufficiently low only on the dorsal side for branching pattern to emerge by diffusion-limited growth.

A further mechanism, similar to the diffusion-limited growth, has been proposed to explain lung branching *in vivo*. Here, however, the authors assume that the FGF concentration on the epithelium is equal everywhere, because the epithelium efficiently absorbs the growth factor [49,54]. What is assumed to differ is the steady-state FGF10 gradient between mesothelium and epithelium. Assuming that FGF10 is uniformly expressed in the submesothelial mesenchyme and FGF receptors are expressed only in the epithelium (such that there is no FGF degradation in the mesenchyme), then the steepness of the steady-state FGF10 gradient between mesothelium and epithelium would depend only on the distance between these tissue layers. If, similar to *in vitro* experiments, some part of the bud was closer to the mesothelium than others, then this part of the bud would experience a steeper FGF10 gradient. If cells could sense gradients rather than sense concentrations, then this could, in principle, drive self-avoiding outgrowth of the branch (figure 9*b*). Moreover, given that the distance between source and sink is key to this mechanism, all buds remain at a comparable distance from the mesothelium during growth. This distance is controlled by the mesenchyme proliferation rate relative to the epithelial proliferation rate. The authors also found that the computed branched tree reproduced multiple morphometric characteristics that have been measured in the adult human lung (i.e. the distribution of branch lengths, diameters and the asymmetry ratio) at least qualitatively. However, in experiments in which FGF10 was added to lung cultures, the mRNA expression of the FGF10 target *Sprouty2* was found to be upregulated [55]. This result is consistent with a model in which cells respond to the FGF10 concentration, but is difficult to explain with a gradient-based mechanism, as the homogeneous addition of FGF10 should reduce the gradient and should thus result in a lower expression level.

We note that stereotyped branching could be obtained with diffusion-limited growth only if there was a preceding mechanism in place to generate the same irregularities in the starting geometry for all buds, so that diffusion-limited growth would be initiated always at the same location to obtain the same overall branching pattern.

#### 3.3.2. Distance-based patterning

In an alternative model, a different distance-dependent effect has been proposed based on the regulatory interactions between SHH and FGF10 [12]. SHH signalling represses *Fgf10* expression, but *Shh* is expressed only in the epithelium, and *Fgf10* is expressed only in the mesenchyme (figure 4*a*). Accordingly, it has been proposed that the inhibitory effect of SHH on *Fgf10* expression will be stronger, the thinner the mesenchyme. Thus, it has been argued that a locally thinner mesenchyme would lead to the local repression of *Fgf10* expression, and thus to the accumulation of FGF10 on the sides, thereby triggering bifurcating outgrowth. Hirashima & Iwasa [11] studied a computational implementation of the model on a static two-dimensional domain in the shape of lung bud cross-section. The model focused on the interactions between FGF10, SHH and TGF-β. According to the model, TGF-β is restricted to the stalk and prevents *Fgf10* expression, whereas SHH is restricted to the tip, and enhances *Fgf10* expression at low concentrations and represses *Fgf10* expression at high concentrations. As a result FGF10 is concentrated at the tip as long as the bud is sufficiently far away that the concentration of SHH is low (figure 9*c*). As the tip grows closer to the impermeable boundary, the local SHH concentration increases and suppresses *Fgf10* expression. As a result, the FGF10 profile splits (figure 9*d*). While the regulatory interactions in the model are plausible, the mesothelium that surrounds the lung bud is unlikely to present a diffusion barrier. In fact, culture experiments show that protein ligands that are added to the lumen of cultured lungs cannot diffuse to the mesenchyme, but protein ligands that are added to the culture medium can pass through the mesothelium to regulate the mesenchyme [56]. Without an impermeable mesothelial boundary, the mechanism does not lead to a bifurcating profile (figure 9*e*) [50]. We note that this distance-based mechanism would also not explain the lateral branching mode. It is therefore likely that a different mechanism controls branching morphogenesis in the lung.

#### 3.3.3. Diffusion-based geometry effect

**Figure 10.**
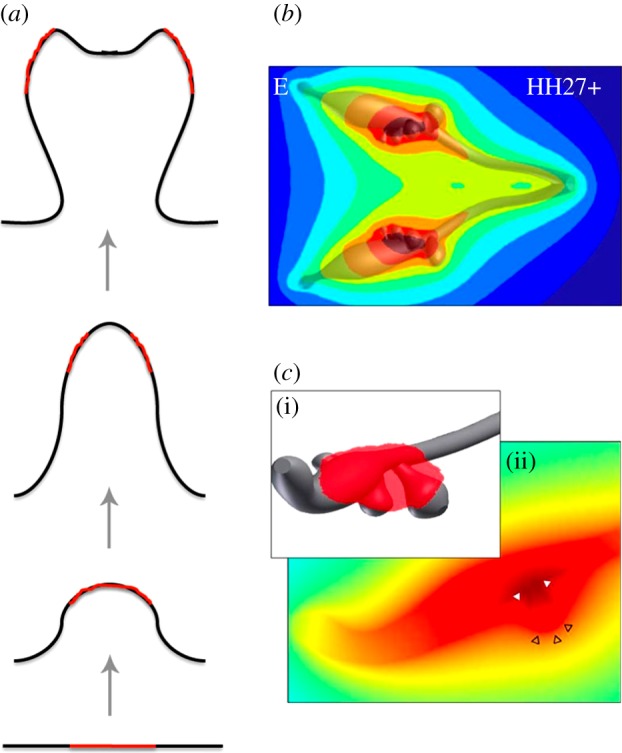
Branching as a result of a diffusion-based geometry effect. (*a*) A cartoon of the proposed geometry-based branching mechanism. As a result of stronger diffusion-based loss at the edges, the concentration of the signal is highest in the centre of the domain (red line), and drives the outgrowth of a bud. As the bud elongates, more signal is lost at the tip than at the sides, because of the higher curvature, and a bifurcating concentration profile of the signalling factor emerges. Computational studies confirm that the geometry effect results in bifurcating concentration profiles, but reveals that it does not support bifurcating outgrowth (D. Menshykau & D. Iber 2013, unpublished data). (*b*) The simulated concentration profile of a ligand that is uniformly secreted from the epithelium of extracted three-dimensional chicken lung bud (Hamburger–Hamilton (HH) stage 27+) into a large computational bounding box [58]. The concentration profiles were normalized to the highest value (red, highest relative concentration; blue, lowest). (*c*) (i) A three-dimensional solid model representation of the region of highest ligand concentration represented by the red shading. (ii) The morphogen concentration in a cross-section through a bud. The bud stalk and branch point have a local maximum (solid white triangles), whereas the bud tip has a local minimum (empty black triangles) in the predicted ligand concentration [58].

*In vitro* experiments demonstrate that the geometry of the domain can affect its patterning [57]. Thus, as a result of diffusion, more signals are lost at the edges of a domain than in its centre, if a signal-producing domain is embedded in a non-producing domain, and the signal can diffuse. As a result of this geometry effect, signal then concentrates in the centre of the domain (figure 10*a*). If this factor supports outgrowth of the tissue, then a bud can form. As the bud elongates, more signal is lost at the tip than at the sides, because of the higher curvature, and a bifurcating concentration profile of the signalling factor emerges. Computational studies not only confirm the emergence of a bifurcating profile as a result of a diffusion-based geometry effect, but also show that it does not support bifurcating outgrowth (D. Menshykau & D. Iber 2013, unpublished data). The geometry effect may nonetheless play an important role in branching morphogenesis by inducing an initial pattern that can then be ‘fixed’ by other processes. When three-dimensional shapes of lung bud epithelia were used in a simulation that had been extracted from early developing chicken lungs, simulated secretion of a ligand from the epithelium into a large computational bounding box (that would model the mesenchyme) resulted in a steady-state concentration pattern that approximately coincided with where the authors would expect branching of secondary bronchi to be inhibited (figure 10*b,c*) [58].

#### 3.3.4. Ligand–receptor-based Turing mechanism

**Figure 11.**
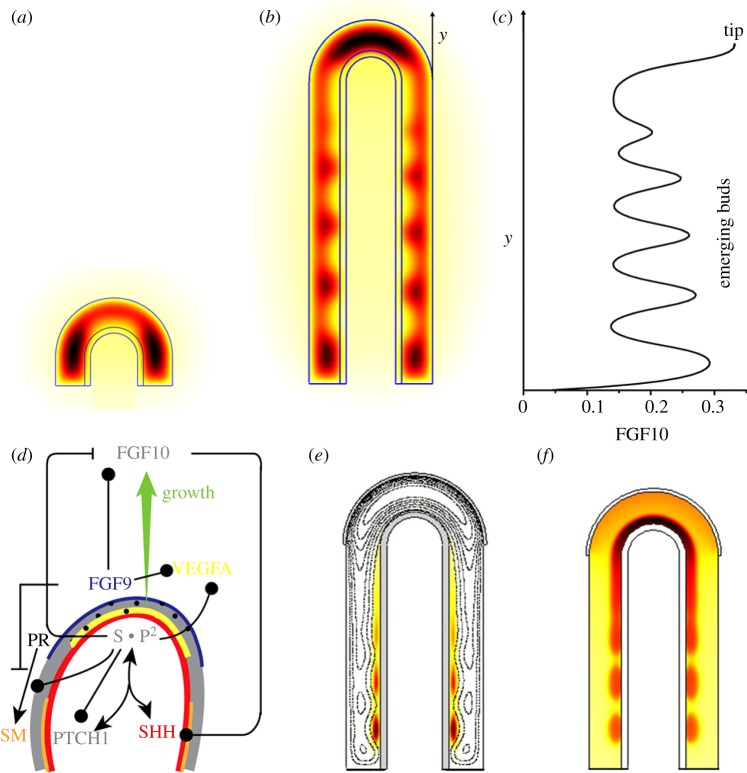
A receptor–ligand-based Turing model for lung branching morphogenesis. (*a,b*) The regulatory network shown in figure 3*f* gives rise to a Turing pattern and results in distributions of FGF10 (colour code: red, highest; black, lowest) and SHH (black and white contour line plot) on bud-shaped domains as characteristic for (*a*) bifurcation or (*b*) lateral branching events. (*c*) The FGF10 concentration profile along the lung bud. (*d*) An extended network that includes also FGF9 also reproduces the observed patterns of smooth muscle (SM) formation from progenitors (PR) and *Vegfa* expression during lung branching morphogenesis. (*e*) Smooth muscles (colour code) emerge in the clefts between lung buds (contour lines mark FGF10 concentration levels) as the lung bud grows out. (*f*) *Vegfa* expression, an inducer of blood vessel formation, emerges in the distal subepithelial mesenchyme. (*a–c*) Adapted from Menshykau *et al.* [50]; (*d–f*) adapted from Celliere *et al.* [60].

We have recently proposed a ligand–receptor-based Turing mechanism to explain the stereotyped branching processes in the lung [50]. We showed that the reported biochemical interactions between FGF10, SHH and PTCH1 (figure 4*a*) give rise to a Turing pattern [59] that yields the FGF10 patterns, which represent the two modes of branching in the developing lung: bifurcation and lateral branching (figure 11*a,b*). The initial model analysis was carried out on a two-dimensional slice of the lung bud. Importantly, the Turing pattern not only permitted the emergence of rings (cylindrical symmetry), but also of spotty patterns on a three-dimensional lung bud shape (rotational symmetry; figure 5). Mutations in genes that are not part of the FGF10/SHH core patterning module have been shown to affect the branching pattern. We find that alterations in almost any parameter value can switch the branching mode in our model. The impact of these other gene products on the branching pattern can thus be explained with indirect effects (i.e. by affecting the parameter values of the core model). Another interesting parameter value is the growth rate. We observed bifurcating patterns at low growth speed and lateral branching patterns at high growth speed. Interestingly, we found that the FGF10 concentration differs in the spots that emerge during lateral branching (figure 11*c*). If the outgrowth speed depended on the FGF10 concentration, then these different concentrations might explain the different branching sequences observed for different branches.

Including FGF9, which is expressed in the distal mesothelium (figure 11*d*, blue line), and which enhances the expression of *Fgf10*, promoted lateral branching over the bifurcation mode of branching as observed in the embryo. Further simulations showed that the expanded core regulatory network was capable of controlling the emergence of smooth muscles in the clefts between growing lung buds (figure 11*e*), and *Vegfa* expression, an inducer of blood vessel formation, in the distal subepithelial mesenchyme (figure 11*f*) [60]. How far the vasculature impacts back on the branching pattern is still a matter of investigation. Thus, while ablation of the vasculature impacted on the three-dimensional branching pattern, in that branches formed parallel or at a shallow angle instead of perpendicular to the axis, the authors noted that ‘inhibition of normal branching resulting from vascular loss could be explained in part by perturbing the unique spatial expression pattern of the key branching mediator *Fgf10* and by misregulated expression of the branching regulators *Shh* and *Sprouty2*’ [61, p. 2359].

Another open question concerns the relative width and length of branches. The proposed Turing mechanism would only explain branch point selection and the choice of the branching mode. How the diameters of the developing bronchial tree are determined relative to the length of each branch element is still an open question. Recent experiments suggest that signalling by extracellular signal-regulated kinase 1 (ERK1) and ERK2, a downstream target of FGF signalling, plays an important role because it affects the cell division plane [62]. A bias in the cell division plane can bias tissue growth in a direction and thus result in either elongation or in a widening of the stalk. Cells that divide parallel to the airway longitudinal axis have lower levels of ERK1/2 signalling, and removal of *Sprouty* genes, which encode negative regulators of FGF10 signalling, results in the random orientation of cell division planes in the stalk and in airways that are wider and shorter than normal [62].

## 4. Kidney branching morphogenesis

Similar to the lung, the kidney collecting ducts form via branching of an epithelial cell layer. During kidney development, the ureteric bud invades the metanephric mesenchyme around embryonic day (E) 10.5 [63]. According to culture experiments, most branching events in the kidney are terminal bifurcations and to a lesser extent trifurcations, and only 6% of all branching events are lateral branching events [4–6]. FGF10 is not necessary for branching in the lung as branching is still observed in *Fgfr2*-mutant mice, though at a reduced rate [64]. Core to the branching mechanism appears to be the TFG-β family protein GDNF. It is expressed in the mesenchyme, and signals via its receptor (RET) and co-receptor GDNF family receptor alpha (Gfrα)1 in the epithelium (figure 4*d*); *Gdnf*, *Ret* and *Gfrα1* null mice do not develop kidneys [6,18–20,63,65,66]. FGF and GDNF signalling appear to cooperate, because activation of FGF10-FGFR2 signalling by knocking out the antagonist *Sprouty* can rescue *Gdnf^–/–^*and *Ret^–/–^* mutants, which otherwise fail to develop kidneys [67]. Moreover, GDNF and FGF10 signalling have, at least in part, the same transcriptional targets [68]. This suggests that the branching mechanisms for lung and kidney are somewhat related in spite of their apparently different molecular nature. An important difference, however, concerns the core feedback structure. While FGF10 and SHH engage in a negative feedback in the lung, GDNF engages in a positive feedback with WNT11 in the ureteric bud [63].

Beads soaked with GDNF induce the outgrowth of extra ureteric buds in kidney culture explants [6,18–20,63,65]. Based on the chemoattractive properties of GDNF [21,69], it was suggested that branching of the ureteric bud is caused by the attraction of the tips towards local sources of GDNF [70]. Accordingly, in early theoretical work, the ureteric bud shape was proposed to be controlled by the interplay of cell proliferation and cell chemotaxis [71]. If chemotaxis towards a source of growth factors (i.e. GDNF) dominates relative to the general growth, then the computed branched structure is kinked, with clearly discernible buds. On the other hand, if growth dominates relative to chemotaxis, then the developing bud is round. The model did not attempt to address the question of how a split expression pattern emerges in the first place. Rather, given the split expression of the signalling protein *Gdnf* (as hard-coded in the model), the model addressed the different possible bud shapes.

**Figure 12.**
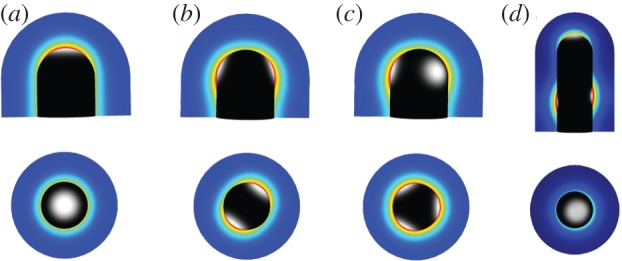
Receptor–ligand-based Turing model for kidney branching morphogenesis. The reported regulatory interactions shown in figure 4*d* can result in self-emerging patterns of the GDNF-bound RET complex in the epithelium (grey scale: white, highest; black, lowest), and *Gdnf* expression in the mesenchyme (colour scale: red, highest; blue, lowest), when solved on a three-dimensional idealized bud-shaped domain. The different patterns can, in principle, (*a*) support elongation, or support the formation of (*b*) bifurcations, (*c*) trifurcations or (*d*) lateral branching. (*a*–*d*) Adapted from Menshykau & Iber [72].

**Figure 13.**
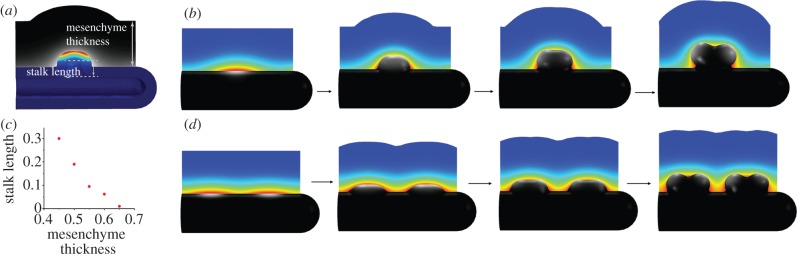
Branching of the ureteric bud into the metanephric mesenchyme. (*a*) The computational domain for the simulation of an ureteric bud after it has started to branch from the Wolffian duct into the metanephric mesenchyme. The double arrows illustrate the stalk length and the mesenchyme thickness. (*b*) The branching of the ureteric bud into the metanephric mesenchyme in response to GDNF signalling. Here, the bud grows out normal to its surface and at a speed proportional to the local concentration of the GDNF-receptor complex, as described by Iber *et al.* [73]. (*c*) The dependency of the length of the stalk to the first branching point on the thickness of the mesenchyme (both as defined in (*a*)). (*d*) The branching of the ureteric bud into the metanephric mesenchyme in a Sprouty^–/–^ mutant. The concentration of the GDNF-bound RET complex in the epithelium is indicated as a grey scale (white, highest; black, lowest); the strength of *Gdnf* expression in the mesenchyme is represented by a colour scale (red, highest; blue, lowest). (*a*–*d*) Adapted from Menshykau & Iber [72].

We have recently developed a three-dimensional model for branching morphogenesis in the kidney [72]. Here we noted that, similar to the lung, the reported biochemical interactions between GDNF and its receptors (figure 4*d*) result in a Turing pattern. Much as reported for the embryo, split concentration patterns, as are characteristic for bifurcations (figure 12*b*) and trifurcations (figure 12*c*), dominate in the model for physiological parameter values, whereas elongation (figure 12*a*) and subsequent lateral branching (figure 12*d*) are rather rare. The patterning mechanism can also support the invasion of the ureteric bud into the metanephric mesenchyme (figure 13*a,b*). It is thus possible that the induction and outgrowth of the ureteric bud from the Wolffian duct works by the same mechanism. Interestingly, in the case of thicker mesenchyme, the length of the outgrowing stalk is shorter and branching happens earlier (figure 13*c*). This is in good agreement with experimental observations in the lung, where side-branching was noted to occur when sufficient space becomes available around the circumference of a parent branch [74].

Turing mechanisms have been proposed for many other biological patterning phenomena, and reproduce the size- and geometry-dependence of biological patterns of various complexity [59,75]. In spite of the great similarity of simulated and real patterns, it remains to be established whether Turing-type mechanisms rather than alternative mechanisms underlie their establishment [76]. In fact, in several cases, Turing-type mechanisms have been wrongly assigned to patterning processes; for example, to explain the stripy expression pattern of pair-rule genes that emerge during *Drosophila* development [77]. These failures reveal the importance of a careful and comprehensive analysis of the underlying molecular interactions. That this is still not always carried out is largely due to the lack of sufficient information regarding the chemical properties of the morphoregulatory proteins (half-life, diffusion rates, endogenous concentrations, activities, etc.) and the molecular nature of their interactions (activating, inhibitory, etc.). Mutant phenotypes, if considered comprehensively rather than selectively, can also provide valuable information to challenge Turing models.

In the case of lung and kidney branching morphogenesis, we showed that, for the identified network components, the Turing model reproduces all available mutant phenotypes. In particular, in the case of lung branching morphogenesis allelic sequences of the *Fgf10* knockdowns are available. Computational modelling showed that the inter-bud (inter-spot) distance is constant as the *Fgf10* expression rate is reduced from 100% to 50%, but it greatly increases as the *Fgf10* expression rate decreases below 50% [60]. This is in perfect agreement with the experimental results that show that WT, *Fgf10*^LacZ/+^ and *Fgf10^+/–^* have normal phenotypes and *Fgf10* expression between 50% and 100%, whereas *Fgf10* expression in *Fgf10*^LacZ/–^ mice is reduced to below 50%, and an abnormal phenotype with an increased distance between branching points is observed [78,79]. For kidney branching morphogenesis, a *Wnt11*/*Ret* allelic series has been reported [63], and, much as in the simulations, the phenotype of *Wnt11* and *Ret* double mutants become more severe as the expression of both *Ret* and *Wnt11* decreases [72]. Finally, in *Sprouty* mutants, several ureteric buds branch from the Wolffian duct, as also observed in the simulation (figure 13*d*).

A receptor–ligand-based Turing mechanism is thus an attractive candidate mechanism for the control of branching morphogenesis in both lung and kidney, in spite of the different proteins involved. In the following, we will compare the core networks that control branching morphogenesis in the glands with those in lung and kidney.

## 5. Branching morphogenesis in glands

### 5.1. Salivary glands

The signalling proteins that regulate branching morphogenesis in the salivary gland are well characterized, and FGF signalling is necessary for branching morphogenesis (figure 4*b*). Thus, transgenic mice lacking FGF8, FGF10, FGFR2b or EGFR do not develop further than the initial bud stage (*Fgf8*-conditional null mice, *Fgf10*-null mice and *Fgfr2b*-null mice), or they have substantially fewer terminal buds (*Egfr*-null mice) [15]. FGF signalling represses *Wnt* expression [80]. WNT signalling upregulates the expression of *Eda* in mesenchymal cells and EDA upregulates *Shh* expression in epithelial cells via NF-kB signalling [81]. SHH signalling upregulates *Fgf8* expression [82], which, in turn, upregulates *Fgf10* and *Shh* expression [83]. Removal of the *Eda receptor* or *Shh* results in dysplasia [82]. Knock-out of *Bmp7* results in reduced branching, and BMP7 is able to rescue branching in salivary glands treated with the FGFR signalling inhibitor SU5402, suggesting that BMP7 may be downstream of FGFR signalling, or in a parallel pathway [84]. The receptor–ligand interactions are thus very similar to those in the lung (e.g. FGF10-FGFR2b or SHH-PTCH1) and kidney (i.e. WNT and FGF10), and mesenchyme from lung buds can induce a branching of cultured submandibular epithelium [32]. Given these parallels, a similar Turing-based mechanism may also control branching in the salivary gland. Experiments in which the FGF10 diffusivity was modulated by alteration of the binding affinity of FGF10 for heparan sulfate highlight the relevance of a diffusion-based mechanism as they reveal an effect on the branching pattern (elongation versus branching) in the salivary gland [14].

### 5.2. Pancreas

In contrast to the classical epithelial budding and tube extension observed in other organs, a pancreatic branch takes shape as a multi-lumen tubular plexus, and coordinately extends and remodels into a ramifying, single-lumen ductal system [85]. This initial tube formation takes place from E9.5 to E12.5. During a secondary transition from E13 to birth, branching morphogenesis is observed [86]. *In vivo* time lapse imaging reveals that the main mode of branching during pancreas branching morphogenesis is lateral branching (86%); terminal bifurcations are also observed, but less often (14%) [87]. The lateral branching mode deployed during pancreas development is simpler than that in the case of lung branching—new buds appear predominantly on one side of the outgrowing tip. However, this might be an artefact of the culture experiment; for example, lung branching patterns are known to be different *in vivo* and *in vitro*. Similar to lung branching morphogenesis, both FGF10/FGFR2b [16,17] and SHH/PTCH1 receptor–ligand modules play an important role during pancreas branching morphogenesis, with Hedgehog signalling repressing *Fgf10* expression (figure 4*c*) [88]. Two factors, BMP4 and FGF10, promote pancreatic morphogenesis at the primary stages of the organogenesis. *Bmp4* is the first to be expressed and promotes budding formation, whereas *Fgf10* is expressed later and promotes (among other things) branching of the pancreatic bud [16]. Ephrin signalling has also been implicated in pancreatic branching as removal of the *EphB2* and *EphB3* receptors results in shortened branches and smaller pancreata [85]. The developing pancreas displays predictable trends in overall shape and in the elaboration of specific branches [85]. The early stages have been simulated using a fully executable, interactive, visual model for four-dimensional simulation of organogenic development [89]. A computational model of the later developmental stages, including the branching morphogenesis of the exocrine pancreas, is still required, as is a model of the processes that result in the differentiation of the pancreatic tissue into ducts and enzyme secreting acinar cells at the tips of the branches.

### 5.3. Prostate

Circulating androgens initiate the development of the prostate from the urogenital sinus [13]. The androgen receptor is expressed in the mesenchyme and is necessary for budding of the urethral epithelium [13]. Androgens have been shown to induce the expression of *Fgf10* and FGF receptor 2 (*Fgfr2*)-IIIb in the urethra [90]. FGF7 and FGF10, expressed in the mesenchyme, bind to FGFR2 on epithelial cells, which leads to the induction and maintenance of *Shh* expression [13]. SHH signalling, in turn, downregulates *Fgf* expression [13]. Moreover, ductal branching and budding are inhibited by the mesenchymal signalling factors BMP4 and BMP7, and stimulated by the antagonist of these BMPs. The regulatory loop is thus very similar to the one in the lung (figure 4*a*), and FGF10 has been found to be essential for the development of the fetal prostate [91]. It should be noted that most of the ducts remain unbranched until birth in rodents, but subsequent epithelial–mesenchymal interactions result in further elongation and branching morphogenesis.

### 5.4. Mammary glands

The development of the mammary proceeds in three stages: a rudimentary gland develops in the embryo and remains quiescent until puberty [92]. During puberty extensive branching occurs, and the mammary glands will undergo further rounds of branching during pregnancy. In male mice, the mesenchyme surrounding the stalk continues to condense until it severs the bud, resulting in a greatly diminished ductal system [93]. Signalling of parathyroid hormone-like hormone (PTHLH) via type 1 PTH/PTHLH receptor (PTH1R) appears to play a key role in the initial process of generating a rudimentary ductal system before birth [94]. *Pthlh* is expressed in the epithelium and signals through its mesenchymal receptor PTH1R to modulate WNT signalling, and to induce the BMP receptor-1A in the mesenchyme [95]. BMP4 signalling can rescue ductal outgrowth in the cultures of *Pthlh^–/–^* mammary buds [95].

Similar to the lung and the kidney, epithelial–mesenchymal signalling by FGFs, EGFs and WNTs plays an important role in controlling branching morphogenesis [92,96]. FGF receptor 2 is required on epithelial cells for ductal elongation [22], but a unique chemoattractant (such as FGF10 in the lung and salivary gland [97] or GDNF in the kidney [21,69]) has not been identified [7]. TGF-β signalling, on the other hand, has been shown to be important to reduce branching, and thereby restrict branch formation [57,98]. TGF-β signalling has been shown to induce the deposition of ECM [57,98] and to affect basal cell proliferation via roundabout 1, SLIT2 and WNT signalling [99]; the non-canonical WNT signalling member WNT5a is necessary for the effects of TGF-β on branching morphogenesis [100].

Culture experiments support a similar control mechanism for branching morphogenesis as in the other organs, as mesenchyme from the salivary gland induces branching of the mammary gland epithelium, even though the branched structure and lobe then resemble that of the salivary gland [101]. Contrary to the lung and the kidney, branching in the mammary glands is, however, not stereotyped [7,96]. The mechanisms that have been proposed to control branching morphogenesis in the mammary gland have so far mainly focused on mechanical constraints, as provided by interactions with the ECM [44,102], and on diffusion-based geometry effects (figure 10*a*) [57,103]. Experiments support an influence of mechanical stress on branching morphogenesis in mammary glands [104], but the composition of the ECM is likely to be the result of regulation by signalling networks [57,98].

## 6. A general mechanism for the control of stereotyped branching

The early branching events differ between the organs (figure 1), but are highly stereotyped (at least in lung and kidney), and must therefore be carefully controlled. A number of mechanisms have been proposed, but most fail to meet all key aspects of such a branching mechanism, which are (i) the production of stereotyped pattern from noisy initial conditions, (ii) pattern stability (or pattern fixation) during outgrowth, such that the pattern can support the outgrowth of the branch, and (iii) the ability to control branching morphogenesis in different organ systems with different signalling networks. The receptor–ligand-based Turing mechanism meets all these conditions [50,60,72]. In particular, it works with a range of signalling networks, because all signalling involves the interaction of secreted ligands with some receptors (figure 4), and any ligand–receptor pair that fulfils the following three conditions can give rise to Turing-type reaction kinetics:

(i) the ligand must diffuse faster than its receptor, which is generally the case for soluble ligands and membrane receptors [105–108];
(ii) receptor and ligand must interact cooperatively, which is typically the case when multimeric components bind each other; and
(iii) receptor–ligand binding must result in increased receptor production. This has been documented in the case of SHH/PTCH1 [109], and GDNF/RET [6,65,68]. In the case of FGF10, both up- and downregulations have been reported [110–113]. BMPs as well as other ligand–receptor systems would also meet the requirements [114,115].

**Figure 14.**
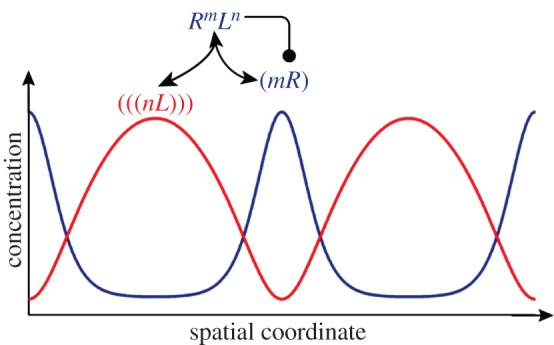
Cooperative receptor–ligand interactions can give rise to Turing patterns. The depicted receptor–ligand interaction can result in spatial patterns via Schnakenberg-type reaction kinetics. Here, *m* receptors (*R*) and *n* ligand molecules (*L*) (with *m* + *n* > 2) need to bind to form the complex *R^m^L^n^*. The receptor–ligand complex then upregulates the receptor concentration (by increasing its expression, limiting its turn-over or similar). To obtain Turing patterns, ligands must diffuse much faster than their receptors. As is characteristic for Schnakenberg-type Turing pattern, the highest receptor and ligand concentrations are then observed in different places.

The ligand–receptor-based Turing mechanism is different from the classical activator–inhibitor Turing mechanism, also known as Gierer–Meinhardt Turing mechanism [116], which is the most commonly studied Turing mechanism. Unlike in the activator–inhibitor mechanism, only one ligand is required in the mechanism proposed here, and the two components (ligand and receptor) assume their highest (and lowest) concentrations in different places (figure 14). The ligand–receptor mechanism is based on Schnakenberg-type reaction kinetics [117], which correspond to the activator-depleted substrate Turing mechanism [116].

Turing mechanisms have been proposed for many systems, and also an alternative Turing-based model has previously been proposed that achieved branching morphogenesis by a combination of patterning and fixation events via cell differentiation [118]. Further experimental studies are now required to carefully test the proposed mechanisms. Turing patterns, much like the other proposed mechanisms, are very sensitive to the geometry of the domain. Simulations of the models on realistic embryonic geometries would therefore present a good test to the model. Developmental sequences of three-dimensional geometries can now be obtained with optical projection tomography (OPT) [119], or two photon or light sheet microscopy [120,121]. Software packages are readily available to construct the four-dimensional geometric series and to efficiently simulate the signalling models on the growing and deforming domains [73,122,123]. The correct mechanism should be able to predict the branch points.

Current models typically consider tissue as a continuum, without cellular resolution. Cell boundaries, however, can have important effects, in particular in the ligand–receptor-based Turing mechanism, where receptor is restricted to the surface of single cells. While we have previously shown that similar patterns can be obtained also on static, cellularized domains [50], it will be important to develop simulation tools that permit the efficient simulation of patterning in three-dimensional cellular systems. In that regard it will be important to include cell growth, diffusion of soluble factors, and diffusion restrictions imposed by cell boundaries to membrane and cytoplasmic proteins.

Finally, it will be important to address the issue that classical Turing models produce patterns only for a tiny parameter space. For such a small parameter space, it is difficult to explain how patterns could emerge in the first place and how they could be preserved during evolution.

## 7. Conclusion

Branching morphogenesis has long fascinated biologists and theoreticians, and many different effects have been defined that all impact on the branching process. We propose that Turing patterns as a result of receptor–ligand interactions constitute a general core mechanism that controls branching morphogenesis in the different organs, as well as other patterning processes in the various developmental systems. Much as complex fractal patterns can be generated with few rules, a Turing mechanism can, in principle, implement a complex, stereotyped sequence of branching events based on few interacting proteins. It should be noted that fractals and Turing patterns are otherwise very different, and Turing mechanisms do not give rise to fractal patterns.

Many of the other discussed effects are likely to affect the patterning process without being its key controller. One such important effect is likely to be the diffusion-based geometry effect (figure 10) [57], which biases the pattern, but which cannot support bifurcating outgrowth by itself. Moreover, the distance between the *Fgf10*-expressing distal mesenchyme (close to the mesothelium) and the *Shh*-expressing epithelium can be expected to affect the exact lung branching pattern without being responsible for the bifurcating concentration profiles *per se* [12]. Similarly, mechanical differences are clearly affecting the branching process. How these arise and how they are controlled in space and time will be an important direction of future research.

A key open question concerns the mechanism that mediates branch outgrowth once an initial symmetry break in the cellular signalling has defined the branch points. This is likely to be the result of a combination of local changes in the proliferation of cells (in particular, their cell division plane), cell deformation and cell migration [62,124,125]. Similarly, it will be of interest to understand how the diameter of the tubes is defined, and how these diameters are shaped to eventually give rise to a fractal-like sequence in the adult.

## References

[1] Affolter M Zeller R Caussinus E 2009 Tissue remodelling through branching morphogenesis. Nat. Rev. Mol. Cell Biol. 10, 831–842 19888266 10.1038/nrm2797

[2] Metzger RJ Klein OD Martin GR Krasnow MA 2008 The branching programme of mouse lung development. Nature 453, 745–750 (doi:10.1038/nature07005) 18463632 10.1038/nature07005 PMC2892995

[3] Blanc P Coste K Pouchin P Azas J-M Blanchon L Gallot D Sapin V 2012 A role for mesenchyme dynamics in mouse lung branching morphogenesis. PLoS ONE 7, e41643 (doi:10.1371/journal.pone.0041643) 22844507 10.1371/journal.pone.0041643 PMC3402475

[4] Watanabe T Costantini F 2004 Real-time analysis of ureteric bud branching morphogenesis *in vitro*. Dev. Biol. 271, 98–108 (doi:10.1016/j.ydbio.2004.03.025) 15196953 10.1016/j.ydbio.2004.03.025

[5] Meyer TN Schwesinger C Bush KT Stuart RO Rose DW Shah MM Vaughn DA Steer DL Nigam SK 2004 Spatiotemporal regulation of morphogenetic molecules during *in vitro* branching of the isolated ureteric bud: toward a model of branching through budding in the developing kidney. Dev. Biol. 275, 44–67 (doi:10.1016/j.ydbio.2004.07.022) 15464572 10.1016/j.ydbio.2004.07.022

[6] Costantini F Kopan R 2010 Patterning a complex organ: branching morphogenesis and nephron segmentation in kidney development. Dev. Cell 18, 698–712 (doi:10.1016/j.devcel.2010.04.008) 20493806 10.1016/j.devcel.2010.04.008 PMC2883254

[7] Ochoa-Espinosa A Affolter M 2012 Branching morphogenesis: from cells to organs and back. Cold Spring Harbor Perspect. Biol. 4, a008243 10.1101/cshperspect.a008243 PMC3475165 22798543

[8] Lu P Werb Z 2008 Patterning mechanisms of branched organs. Science 322, 1506–1509 (doi:10.1126/science.1162783) 19056977 10.1126/science.1162783 PMC2645229

[9] Davies J 2005 Branching morphogenesis. New York, NY: Springer

[10] Harrison LG 2010 The shaping of life. Cambridge, UK: Cambridge University Press

[11] Hirashima T Iwasa Y 2009 Mechanisms for split localization of Fgf10 expression in early lung development. Dev. Dyn. 238, 2813–2822 19842186 10.1002/dvdy.22108

[12] Bellusci S Grindley J Emoto H Itoh N Hogan BL 1997 Fibroblast growth factor 10 (FGF10) and branching morphogenesis in the embryonic mouse lung. Development (Camb. Engl.) 124, 4867–4878 10.1242/dev.124.23.4867 9428423

[13] Wilhelm D Koopman P 2006 The makings of maleness: towards an integrated view of male sexual development. Nat. Rev. Genet. 7, 620–631 (doi:10.1038/nrg1903) 16832429 10.1038/nrg1903

[14] Makarenkova HP 2009 Differential interactions of FGFs with heparan sulfate control gradient formation and branching morphogenesis. Sci. Signal. 2, ra55 (doi:10.1126/scisignal.2000304) 19755711 10.1126/scisignal.2000304 PMC2884999

[15] Hsu JC-F Yamada KM 2010 Salivary gland branching morphogenesis: recent progress and future opportunities. Int. J. Oral Sci. 2, 117–126 (doi:10.4248/IJOS10042) 21125789 10.4248/IJOS10042 PMC3168569

[16] Bhushan A Itoh N Kato S Thiery JP Czernichow P Bellusci S Scharfmann R 2001 Fgf10 is essential for maintaining the proliferative capacity of epithelial progenitor cells during early pancreatic organogenesis. Development (Camb. Engl.) 128, 5109–5117 10.1242/dev.128.24.5109 11748146

[17] Pulkkinen M-A Spencer-Dene B Dickson C Otonkoski T 2003 The IIIb isoform of fibroblast growth factor receptor 2 is required for proper growth and branching of pancreatic ductal epithelium but not for differentiation of exocrine or endocrine cells. Mech. Dev. 120, 167–175 (doi:10.1016/S0925-4773(02)00440-9) 12559489 10.1016/s0925-4773(02)00440-9

[18] Treanor JJ 1996 Characterization of a multicomponent receptor for GDNF. Nature 382, 80–83 (doi:10.1038/382080a0) 8657309 10.1038/382080a0

[19] Pichel JG 1996 Defects in enteric innervation and kidney development in mice lacking GDNF. Nature 382, 73–76 (doi:10.1038/382073a0) 8657307 10.1038/382073a0

[20] Sánchez MP Silos-Santiago I Frisén J He B Lira SA Barbacid M 1996 Renal agenesis and the absence of enteric neurons in mice lacking GDNF. Nature 382, 70–73 (doi:10.1038/382070a0) 8657306 10.1038/382070a0

[21] Tang MJ Worley D Sanicola M Dressler GR 1998 The RET-glial cell-derived neurotrophic factor (GDNF) pathway stimulates migration and chemoattraction of epithelial cells. J. Cell Biol. 142, 1337–1345 (doi:10.1083/jcb.142.5.1337) 9732293 10.1083/jcb.142.5.1337 PMC2149344

[22] Lu P Ewald AJ Martin GR Werb Z 2008 Genetic mosaic analysis reveals FGF receptor 2 function in terminal end buds during mammary gland branching morphogenesis. Dev. Biol. 321, 77–87 (doi:10.1016/j.ydbio.2008.06.005) 18585375 10.1016/j.ydbio.2008.06.005 PMC2582391

[23] Li R Bowerman B 2010 Symmetry breaking in biology. Cold Spring Harbor Perspect. Biol. 2, a003475 (doi:10.1101/cshperspect.a003475) 10.1101/cshperspect.a003475 PMC2829966 20300216

[24] Kitaoka H Takaki R Suki B 1999 A three-dimensional model of the human airway tree. J. Appl. Physiol. 87, 2207–2217 10601169 10.1152/jappl.1999.87.6.2207

[25] West BJ Bhargava V Goldberger AL 1986 Beyond the principle of similitude: renormalization in the bronchial tree. J. Appl. Physiol. 60, 1089–1097 3957825 10.1152/jappl.1986.60.3.1089

[26] Nelson TR West BJ Goldberger AL 1990 The fractal lung: universal and species-related scaling patterns. Experientia 46, 251–254 (doi:10.1007/BF01951755) 2311717 10.1007/BF01951755

[27] Wilson TA 1967 Design of the bronchial tree. Nature 213, 668–669 (doi:10.1038/213668a0) 6031769 10.1038/213668a0

[28] Bleuer BH 1988 Morphometry of the human pulmonary acinus. Anat. Rec. 4, 401–414 10.1002/ar.1092200410 3382030

[29] Panico J Sterling P 1995 Retinal neurons and vessels are not fractal but space-filling. J. Comp. Neurol. 361, 479–490 (doi:10.1002/cne.903610311) 8550894 10.1002/cne.903610311

[30] Mauroy B Filoche M Weibel ER Sapoval B 2004 An optimal bronchial tree may be dangerous. Nature 427, 633–636 (doi:10.1038/nature02287) 14961120 10.1038/nature02287

[31] Lin Y Zhang S Rehn M Itäranta P Tuukkanen J Heljäsvaara R Peltoketo H Pihlajaniemi T Vainio S 2001 Induced repatterning of type XVIII collagen expression in ureter bud from kidney to lung type: association with sonic hedgehog and ectopic surfactant protein C. Development (Camb. Engl.) 128, 1573–1585 10.1242/dev.128.9.1573 11290296

[32] Nogawa H Nakanishi Y 1987 Mechanical aspects of the mesenchymal influence on epithelial branching morphogenesis of mouse salivary gland. Development (Camb. Engl.) 101, 491–500

[33] Lubkin S Murray J 1995 A mechanism for early branching in lung morphogenesis. J. Math. Biol. 34, 77–94 (doi:10.1007/BF00180137) 8568422 10.1007/BF00180137

[34] Jha B Cueto-Felgueroso L Juanes R 2011 Quantifying mixing in viscously unstable porous media flows. Phys. Rev. E 84, 066312 10.1103/PhysRevE.84.066312 22304195

[35] Unbekandt M del Moral P-M Sala FG Bellusci S Warburton D Fleury V 2008 Tracheal occlusion increases the rate of epithelial branching of embryonic mouse lung via the FGF10-FGFR2b-Sprouty2 pathway. Mech. Dev. 125, 314–324 (doi:10.1016/j.mod.2007.10.013) 18082381 10.1016/j.mod.2007.10.013 PMC2275719

[36] Maher JV 1985 Development of viscous fingering patterns. Phys. Rev. Lett. 54, 1498–1501 (doi:10.1103/PhysRevLett.54.1498) 10031054 10.1103/PhysRevLett.54.1498

[37] Wan X Li Z Lubkin SR 2008 Mechanics of mesenchymal contribution to clefting force in branching morphogenesis. Biomech. Model. Mechanobiol. 7, 417–426 (doi:10.1007/s10237-007-0105-y) 17901991 10.1007/s10237-007-0105-y

[38] Lubkin SR 2008 Branched organs: mechanics of morphogenesis by multiple mechanisms. Curr. Top. Dev. Biol. 81, 249–268 (doi:10.1016/S0070-2153(07)81008-8) 18023730 10.1016/S0070-2153(07)81008-8

[39] Lubkin S Li Z 2002 Force and deformation on branching rudiments: cleaving between hypotheses. Biomech. Model. Mechanobiol. 1, 5–16 (doi:10.1007/s10237-002-0001-4) 14586703 10.1007/s10237-002-0001-4

[40] Nogawa H Ito T 1995 Branching morphogenesis of embryonic mouse lung epithelium in mesenchyme-free culture. Development (Camb. Engl.) 121, 1015–1022 10.1242/dev.121.4.1015 7538066

[41] Warburton D 2010 Lung organogenesis. Curr. Top. Dev. Biol. 90, 73–158 (doi:10.1016/S0070-2153(10)90003-3) 20691848 10.1016/S0070-2153(10)90003-3 PMC3340128

[42] Moore KA Polte T Huang S Shi B Alsberg E Sunday ME Ingber DE 2005 Control of basement membrane remodeling and epithelial branching morphogenesis in embryonic lung by Rho and cytoskeletal tension. Dev. Dyn. 232, 268–281 (doi:10.1002/dvdy.20237) 15614768 10.1002/dvdy.20237

[43] Moore TM Shirah WB Khimenko PL Paisley P Lausch RN Taylor AE 2002 Involvement of CD40-CD40L signaling in postischemic lung injury. Am. J. Physiol. Lung Cell. Mol. Physiol. 283, L1255–L1262 12388354 10.1152/ajplung.00016.2002

[44] Kim HY Nelson CM 2012 Extracellular matrix and cytoskeletal dynamics during branching morphogenesis. Organogenesis 8, 56–64 (doi:10.4161/org.19813) 22609561 10.4161/org.19813 PMC3429513

[45] Min H Danilenko DM Scully SA Bolon B Ring BD Tarpley JE DeRose M Simonet WS 1998 Fgf-10 is required for both limb and lung development and exhibits striking functional similarity to *Drosophila* branchless. Genes Dev. 12, 3156–3161 (doi:10.1101/gad.12.20.3156) 9784490 10.1101/gad.12.20.3156 PMC317210

[46] Sekine K 1999 Fgf10 is essential for limb and lung formation. Nat. Genet. 21, 138–141 (doi:10.1038/5096) 9916808 10.1038/5096

[47] Pepicelli CV Lewis PM McMahon AP 1998 Sonic hedgehog regulates branching morphogenesis in the mammalian lung. Curr. Biol. 8, 1083–1086 (doi:10.1016/S0960-9822(98)70446-4) 9768363 10.1016/s0960-9822(98)70446-4

[48] Hartmann D Miura T 2006 Modelling *in vitro* lung branching morphogenesis during development. J. Theor. Biol. 242, 862–872 (doi:10.1016/j.jtbi.2006.05.009) 16808929 10.1016/j.jtbi.2006.05.009

[49] Clément R Blanc P Mauroy B Sapin V Douady S 2012 Shape self-regulation in early lung morphogenesis. PLoS ONE 7, e36925 (doi:10.1371/journal.pone.0036925) 22615846 10.1371/journal.pone.0036925 PMC3353953

[50] Menshykau D Kraemer C Iber D 2012 Branch mode selection during early lung development. PLoS Comput. Biol. 8, e1002377 (doi:10.1371/journal.pcbi.1002377) 22359491 10.1371/journal.pcbi.1002377 PMC3280966

[51] Hartmann D Miura T 2007 Mathematical analysis of a free-boundary model for lung branching morphogenesis. Math. Med. Biol. 24, 209–224 (doi:10.1093/imammb/dql029) 17132681 10.1093/imammb/dql029

[52] Miura T Shiota K 2002 Depletion of FGF acts as a lateral inhibitory factor in lung branching morphogenesis *in vitro*. Mech. Dev. 116, 29–38 (doi:10.1016/S0925-4773(02)00132-6) 12128203 10.1016/s0925-4773(02)00132-6

[53] Miura T Hartmann D Kinboshi M Komada M Ishibashi M Shiota K 2009 The cyst-branch difference in developing chick lung results from a different morphogen diffusion coefficient. Mech. Dev. 126, 160–172 (doi:10.1016/j.mod.2008.11.006) 19073251 10.1016/j.mod.2008.11.006

[54] Clément R Douady S Mauroy B 2012 Branching geometry induced by lung self-regulated growth. Phys. Biol. 9, 066006 (doi:10.1088/1478-3975/9/6/066006) 23160420 10.1088/1478-3975/9/6/066006

[55] Mailleux AA Tefft D Ndiaye D Itoh N Thiery JP Warburton D Bellusci S 2001 Evidence that SPROUTY2 functions as an inhibitor of mouse embryonic lung growth and morphogenesis. Mech. Dev. 102, 81–94 (doi:10.1016/S0925-4773(01)00286-6) 11287183 10.1016/s0925-4773(01)00286-6

[56] Bragg AD Moses HL Serra R 2001 Signaling to the epithelium is not sufficient to mediate all of the effects of transforming growth factor beta and bone morphogenetic protein 4 on murine embryonic lung development. Mech. Dev. 109, 13–26 (doi:10.1016/S0925-4773(01)00508-1) 11677049 10.1016/s0925-4773(01)00508-1

[57] Nelson CM Vanduijn MM Inman JL Fletcher DA Bissell MJ 2006 Tissue geometry determines sites of mammary branching morphogenesis in organotypic cultures. Science 314, 298–300 (doi:10.1126/science.1131000) 17038622 10.1126/science.1131000 PMC2933179

[58] Gleghorn JP Kwak J Pavlovich AL Nelson CM 2012 Inhibitory morphogens and monopodial branching of the embryonic chicken lung. Dev. Dyn. 241, 852–862 22410853 10.1002/dvdy.23771 PMC3443594

[59] Murray JD 2003 Mathematical biology, 3rd edn, vol. 2 *Mathematical biology: II. Spatial models and biomedical applications* New York, NY: Springer

[60] Celliere G Menshykau D Iber D 2012 Simulations demonstrate a simple network to be sufficient to control branch point selection, smooth muscle and vasculature formation during lung branching morphogenesis. Biol. Open 1, 775–788 23213471 10.1242/bio.20121339 PMC3507219

[61] Lazarus A del Moral P-M Ilovich O Mishani E Warburton D Keshet E 2011 A perfusion-independent role of blood vessels in determining branching stereotypy of lung airways. Development (Camb. Engl.) 138, 2359–2368 (doi:10.1242/dev.060723) 10.1242/dev.060723 PMC3091498 21558382

[62] Tang N Marshall WF McMahon M Metzger RJ Martin GR 2011 Control of mitotic spindle angle by the RAS-regulated ERK1/2 pathway determines lung tube shape. Science 333, 342–345 (doi:10.1126/science.1204831) 21764747 10.1126/science.1204831 PMC4260627

[63] Majumdar A Vainio S Kispert A McMahon J McMahon AP 2003 Wnt11 and Ret/Gdnf pathways cooperate in regulating ureteric branching during metanephric kidney development. Development (Camb. Engl.) 130, 3175–3185 (doi:10.1242/dev.00520) 10.1242/dev.00520 12783789

[64] Sims-Lucas S Argyropoulos C Kish K McHugh K Bertram JF Quigley R Bates CM 2009 Three-dimensional imaging reveals ureteric and mesenchymal defects in Fgfr2-mutant kidneys. J. Am. Soc. Nephrol. 20, 2525–2533 (doi:10.1681/ASN.2009050532) 19833900 10.1681/ASN.2009050532 PMC2794230

[65] Pepicelli C Kispert A Rowitch D McMahon A 1997 GDNF induces branching and increased cell proliferation in the ureter of the mouse. Dev. Biol. 192, 193–198 (doi:10.1006/dbio.1997.8745) 9405108 10.1006/dbio.1997.8745

[66] Schuchardt A D'Agati V Larsson-Blomberg L Costantini F Pachnis V 1994 Defects in the kidney and enteric nervous system of mice lacking the tyrosine kinase receptor Ret. Nature 367, 380–383 (doi:10.1038/367380a0) 8114940 10.1038/367380a0

[67] Michos O Cebrian C Hyink D Grieshammer U Williams L D'Agati V Licht JD Martin GR Costantini F 2010 Kidney development in the absence of Gdnf and Spry1 requires Fgf10. PLoS Genet. 6, e1000809 (doi:10.1371/journal.pgen.1000809) 20084103 10.1371/journal.pgen.1000809 PMC2797609

[68] Lu BC 2009 Etv4 and Etv5 are required downstream of GDNF and Ret for kidney branching morphogenesis. Nat. Genet. 41, 1295–1302 (doi:10.1038/ng.476) 19898483 10.1038/ng.476 PMC2787691

[69] Tang M-J Cai Y Tsai S-J Wang Y-K Dressler GR 2002 Ureteric bud outgrowth in response to RET activation is mediated by phosphatidylinositol 3-kinase. Dev. Biol. 243, 128–136 (doi:10.1006/dbio.2001.0557) 11846482 10.1006/dbio.2001.0557

[70] Sariola H Saarma M 2003 Novel functions and signalling pathways for GDNF. J. Cell Sci. 116, 3855–3862 (doi:10.1242/jcs.00786) 12953054 10.1242/jcs.00786

[71] Hirashima T Iwasa Y Morishita Y 2009 Dynamic modeling of branching morphogenesis of ureteric bud in early kidney development. J. Theor. Biol. 259, 58–66 (doi:10.1016/j.jtbi.2009.03.017) 19324059 10.1016/j.jtbi.2009.03.017

[72] Menshykau D Iber D 2013 Kidney branching morphogenesis under the control of a ligand–receptor based Turing mechanism. Phys. Biol. 10, 046003 (doi:10.1088/1478-3975/10/4/046003) 23770927 10.1088/1478-3975/10/4/046003

[73] Iber D Tanaka S Fried P Germann P Menshykau D In press. Simulating tissue morphogenesis and signaling. In Tissue morphogenesis: methods and protocols (ed. Nelson CM). Methods in Molecular Biology New York, NY: Springer 10.1007/978-1-4939-1164-6_21 25245703

[74] Blanc P Coste K Pouchin P Azaïs J-M Blanchon L Gallot D Sapin V 2012 A role for mesenchyme dynamics in mouse lung branching morphogenesis. PLoS ONE 7, e41643 (doi:10.1371/journal.pone.0041643) 22844507 10.1371/journal.pone.0041643 PMC3402475

[75] Kondo S Miura T 2010 Reaction–diffusion model as a framework for understanding biological pattern formation. Science 329, 1616–1620 (doi:10.1126/science.1179047) 20929839 10.1126/science.1179047

[76] Hoefer T Maini P 1996 Turing patterns in fish skin? Nature 380, 678 (doi:10.1038/380678a0)

[77] Akam M 1989 *Drosophila* development: making stripes inelegantly. Nature 341, 282–283 (doi:10.1038/341282a0) 2797143 10.1038/341282a0

[78] Mailleux AA 2005 Fgf10 expression identifies parabronchial smooth muscle cell progenitors and is required for their entry into the smooth muscle cell lineage. Development 132, 2157–2166 (doi:10.1242/dev.01795) 15800000 10.1242/dev.01795

[79] Ramasamy SK 2007 Fgf10 dosage is critical for the amplification of epithelial cell progenitors and for the formation of multiple mesenchymal lineages during lung development. Dev. Biol. 307, 237–247 (doi:10.1016/j.ydbio.2007.04.033) 17560563 10.1016/j.ydbio.2007.04.033 PMC3714306

[80] Larsen M Yamada KM Musselmann K 2010 Systems analysis of salivary gland development and disease. Wiley Interdiscip. Rev. Syst. Biol. Med. 2, 670–682 (doi:10.1002/wsbm.94) 20890964 10.1002/wsbm.94 PMC3398465

[81] Häärä O Fujimori S Schmidt-Ullrich R Hartmann C Thesleff I Mikkola ML 2011 Ectodysplasin and Wnt pathways are required for salivary gland branching morphogenesis. Development (Camb. Engl.) 138, 2681–2691 (doi:10.1242/dev.057711) 10.1242/dev.057711 21652647

[82] Jaskoll T Leo T Witcher D Ormestad M Astorga J Bringas P Carlsson P Melnick M 2004 Sonic hedgehog signaling plays an essential role during embryonic salivary gland epithelial branching morphogenesis. Dev. Dyn. 229, 722–732 (doi:10.1002/dvdy.10472) 15042696 10.1002/dvdy.10472

[83] Jaskoll T Witcher D Toreno L Bringas P Moon AM Melnick M 2004 FGF8 dose-dependent regulation of embryonic submandibular salivary gland morphogenesis. Dev. Biol. 268, 457–469 (doi:10.1016/j.ydbio.2004.01.004) 15063181 10.1016/j.ydbio.2004.01.004

[84] Patel VN Rebustini IT Hoffman MP 2006 Salivary gland branching morphogenesis. Differentiation 74, 349–364 (doi:10.1111/j.1432-0436.2006.00088.x) 16916374 10.1111/j.1432-0436.2006.00088.x

[85] Villasenor A Chong DC Henkemeyer M Cleaver O 2010 Epithelial dynamics of pancreatic branching morphogenesis. Development (Camb. Engl.) 137, 4295–4305 (doi:10.1242/dev.052993) 10.1242/dev.052993 PMC2990215 21098570

[86] Benitez CM Goodyer WR Kim SK 2012 Deconstructing pancreas developmental biology. Cold Spring Harbor Perspect. Biol. 4, a012401 (doi:10.1101/cshperspect.a012401) 10.1101/cshperspect.a012401 PMC3367550 22587935

[87] Puri S Hebrok M 2007 Dynamics of embryonic pancreas development using real-time imaging. Dev. Biol. 306, 82–93 (doi:10.1016/j.ydbio.2007.03.003) 17448459 10.1016/j.ydbio.2007.03.003 PMC1988845

[88] Kawahira H Ma NH Tzanakakis ES McMahon AP Chuang P-T Hebrok M 2003 Combined activities of hedgehog signaling inhibitors regulate pancreas development. Development (Camb. Engl.) 130, 4871–4879 (doi:10.1242/dev.00653) 10.1242/dev.00653 12917290

[89] Setty Y Cohen IR Dor Y Harel D 2008 Four-dimensional realistic modeling of pancreatic organogenesis. Proc. Natl Acad. Sci. USA 105, 20 374–20 379 (doi:10.1073/pnas.0808725105) 19091945 10.1073/pnas.0808725105 PMC2629264

[90] Petiot A Perriton CL Dickson C Cohn MJ 2005 Development of the mammalian urethra is controlled by Fgfr2-IIIb. Development (Camb. Engl.) 132, 2441–2450 (doi:10.1242/dev.01778) 10.1242/dev.01778 PMC12904671 15843416

[91] Donjacour AA Thomson AA Cunha GR 2003 FGF-10 plays an essential role in the growth of the fetal prostate. Dev. Biol. 261, 39–54 (doi:10.1016/S0012-1606(03)00250-1) 12941620 10.1016/s0012-1606(03)00250-1

[92] Macias H Hinck L 2012 Mammary gland development. Wiley Interdiscip. Rev. Dev. Biol. 1, 533–557 (doi:10.1002/wdev.35) 22844349 10.1002/wdev.35 PMC3404495

[93] Kratochwil K Schwartz P 1976 Tissue interaction in androgen response of embryonic mammary rudiment of mouse: identification of target tissue for testosterone. Proc. Natl Acad. Sci. USA 73, 4041–4044 (doi:10.1073/pnas.73.11.4041) 1069291 10.1073/pnas.73.11.4041 PMC431320

[94] Wysolmerski JJ Philbrick WM Dunbar ME Lanske B Kronenberg H Broadus AE 1998 Rescue of the parathyroid hormone-related protein knockout mouse demonstrates that parathyroid hormone-related protein is essential for mammary gland development. Development (Camb. Engl.) 125, 1285–1294 10.1242/dev.125.7.1285 9477327

[95] Hens JR Dann P Zhang J-P Harris S Robinson GW Wysolmerski J 2007 BMP4 and PTHrP interact to stimulate ductal outgrowth during embryonic mammary development and to inhibit hair follicle induction. Development (Camb. Engl.) 134, 1221–1230 (doi:10.1242/dev.000182) 10.1242/dev.000182 17301089

[96] Gjorevski N Nelson CM 2011 Integrated morphodynamic signalling of the mammary gland. Nat. Rev. Mol. Cell Biol. 12, 581–593 (doi:10.1038/nrm3168) 21829222 10.1038/nrm3168

[97] Park WY Miranda B Lebeche D Hashimoto G Cardoso WV 1998 FGF-10 is a chemotactic factor for distal epithelial buds during lung development. Dev. Biol. 201, 125–134 (doi:10.1006/dbio.1998.8994) 9740653 10.1006/dbio.1998.8994

[98] Pierce DF Johnson MD Matsui Y Robinson SD Gold LI Purchio AF Daniel CW Hogan BL Moses HL 1993 Inhibition of mammary duct development but not alveolar outgrowth during pregnancy in transgenic mice expressing active TGF-β 1. Genes Dev. 7, 2308–2317 (doi:10.1101/gad.7.12a.2308) 8253379 10.1101/gad.7.12a.2308

[99] Macias H Moran A Samara Y Moreno M Compton JE Harburg G Strickland P Hinck L 2011 SLIT/ROBO1 signaling suppresses mammary branching morphogenesis by limiting basal cell number. Dev. Cell 20, 827–840 (doi:10.1016/j.devcel.2011.05.012) 21664580 10.1016/j.devcel.2011.05.012 PMC3129866

[100] Roarty K Serra R 2007 Wnt5a is required for proper mammary gland development and TGF-β-mediated inhibition of ductal growth. Development (Camb. Engl.) 134, 3929–3939 (doi:10.1242/dev.008250) 10.1242/dev.008250 17898001

[101] Kratochwil K 1969 Organ specificity in mesenchymal induction demonstrated in the embryonic development of the mammary gland of the mouse. Dev. Biol. 20, 46–71 (doi:10.1016/0012-1606(69)90004-9) 5795848 10.1016/0012-1606(69)90004-9

[102] Pozzi A Zent R 2011 Extracellular matrix receptors in branched organs. Curr. Opin. Cell Biol. 23, 547–553 (doi:10.1016/j.ceb.2011.04.003) 21561755 10.1016/j.ceb.2011.04.003 PMC3181278

[103] Nelson CM 2012 Symmetry breaking during morphogenesis in the embryo and in engineered tissues. AIChE J. 58, 3608–3613 (doi:10.1002/aic.13941)

[104] Gjorevski N Nelson CM 2010 The mechanics of development: Models and methods for tissue morphogenesis. Birth Defects Res. C, Embryo Today Rev. 90, 193–202 (doi:10.1002/bdrc.20185) 10.1002/bdrc.20185 PMC3087175 20860059

[105] Choquet D Triller A 2003 The role of receptor diffusion in the organization of the postsynaptic membrane. Nat. Rev. Neurosci. 4, 251–265 (doi:10.1038/nrn1077) 12671642 10.1038/nrn1077

[106] Ries J Yu SR Burkhardt M Brand M Schwille P 2009 Modular scanning FCS quantifies receptor–ligand interactions in living multicellular organisms. Nat. Methods 6, 643–645 (doi:10.1038/nmeth.1355) 19648917 10.1038/nmeth.1355

[107] Kumar M Mommer MS Sourjik V 2010 Mobility of cytoplasmic, membrane, DNA-binding proteins in *Escherichia coli*. Biophys. J. 98, 552–559 (doi:10.1016/j.bpj.2009.11.002) 20159151 10.1016/j.bpj.2009.11.002 PMC2820653

[108] Hebert B Costantino S Wiseman P 2005 Spatiotemporal image correlation spectroscopy (STICS) theory, verification, application to protein velocity mapping in living CHO cells. Biophys. J. 88, 3601–3614 (doi:10.1529/biophysj.104.054874) 15722439 10.1529/biophysj.104.054874 PMC1305507

[109] Weaver M Batts L Hogan B 2010 Tissue interactions pattern the mesenchyme of the embryonic mouse lung. Dev. Biol. 258, 169–184 (doi:10.1016/S0012-1606(03)00117-9) 12781691 10.1016/s0012-1606(03)00117-9

[110] Bansal R Pfeiffer S 1997 Regulation of oligodendrocyte differentiation by fibroblast growth factors. Adv. Exp. Med. Biol. 429, 69–77 (doi:10.1007/978-1-4757-9551-6_5) 9413566 10.1007/978-1-4757-9551-6_5

[111] Estival A Monzat V Miquel K Gaubert F Hollande E Korc M Vaysse N Clementem F 2010 Differential regulation of fibroblast growth factor (fgf) receptor-1 mrna and protein by two molecular forms of basic fgf. Modulation of fgfr-1 mrna stability. Dev. Cell 18, 698–712 8621430 10.1074/jbc.271.10.5663

[112] Ota S Tonou-Fujimori N Tonou-Fujimori N Nakayama Y Ito Y Kawamura A Yamasu K 2010 Fgf receptor gene expression and its regulation by fgf signaling during early zebrafish development. Genesis 48, 707–716 (doi:10.1002/dvg.20682) 20960516 10.1002/dvg.20682

[113] Zakrzewska M Haugsten EM Nadratowska-Wesolowska B Oppelt A Hausott B Jin Y Otlewski J Wesche J Wiedlocha A 2013 ERK-mediated phosphorylation of fibroblast growth factor receptor 1 on Ser777 inhibits signaling. Sci. Signal. 6, ra11 (doi:10.1126/scisignal.2003087) 23405013 10.1126/scisignal.2003087

[114] Merino R Gañan Y Macias D Economides AN Sampath KT Hurle JM 1998 Morphogenesis of digits in the avian limb is controlled by FGFs, TGFbetas, noggin through BMP signaling. Dev. Biol. 200, 35–45 (doi:10.1006/dbio.1998.8946) 9698454 10.1006/dbio.1998.8946

[115] Badugu A Kraemer C Germann P Menshykau D Iber D 2012 Digit patterning during limb development as a result of the BMP-receptor interaction. Sci. Rep. 2, 991 (doi:10.1038/srep00991) 23251777 10.1038/srep00991 PMC3524521

[116] Gierer A Meinhardt H 1972 A theory of biological pattern formation. Kybernetik 12, 30–39 (doi:10.1007/BF00289234) 4663624 10.1007/BF00289234

[117] Schnakenberg J 1979 Simple chemical reaction systems with limit cycle behaviour. J. Theor. Biol. 81, 389–400 (doi:10.1016/0022-5193(79)90042-0) 537379 10.1016/0022-5193(79)90042-0

[118] Meinhardt H 1976 Morphogenesis of lines and nets. Differ. Res. Biol. Divers. 6, 117–123 (doi:10.1111/j.1432-0436.1976.tb01478.x) 10.1111/j.1432-0436.1976.tb01478.x 1010155

[119] Sharpe J Ahlgren U Perry P Hill B Ross A Jacob Hecksher-Sorensen RB Davidson D 2002 Optical projection tomography as a tool for 3D microscopy and gene expression studies. Science 296, 541–545 (doi:10.1126/science.1068206) 11964482 10.1126/science.1068206

[120] Verveer PJ Swoger J Pampaloni F Greger K Marcello M Stelzer EHK 2007 High-resolution three-dimensional imaging of large specimens with light sheet-based microscopy. Nat. Methods 4, 311–313 17339847 10.1038/nmeth1017

[121] Truong TV Supatto W Koos DS Choi JM Fraser SE 2011 Deep and fast live imaging with two-photon scanned light-sheet microscopy. Nat. Methods 8, 757–760 (doi:10.1038/nmeth.1652) 21765409 10.1038/nmeth.1652

[122] Menshykau D Iber D 2012 Simulating organogenesis with Comsol: interacting and deforming domains. *Proceedings of COMSOL Conf. 2012*. See http://arxiv.org/abs/1210.0810

[123] Germann P Menshykau D Tanaka S Iber D 2011 Simulating organogensis in Comsol. In *Proceedings of COMSOL Conference 2011*. See http://arxiv.org/abs/1210.0428

[124] Kim HY Varner VD Nelson CM 2013 Apical constriction initiates new bud formation during monopodial branching of the embryonic chicken lung. Development (Camb. Engl.) 140, 3146–3155 10.1242/dev.093682 PMC3931740 23824575

[125] Schnatwinkel C Niswander L 2013 Multiparametric image analysis of lung-branching morphogenesis. Dev. Dyn. 242, 622–637 (doi:10.1002/dvdy.23961) 23483685 10.1002/dvdy.23961 PMC3833092

